# Renal scintigraphy to predict persistent renal failure after acute kidney injury: an observational study

**DOI:** 10.1007/s40620-023-01569-0

**Published:** 2023-02-02

**Authors:** Marco Altarelli, Mario Jreige, John Olivier Prior, Marie Nicod Lalonde, Antoine Guillaume Schneider

**Affiliations:** 1 Adult Intensive Care Unit, Centre Hospitalier Universitaire Vaudois (CHUV), 46, Avenue du Bugnon, 1011 Lausanne, Switzerland; 2 Nuclear Medicine and Molecular Imaging, Centre Hospitalier Universitaire Vaudois (CHUV), Lausanne, Switzerland; 3 Faculty of Biology and Medicine, University of Lausanne, Lausanne, Switzerland

**Keywords:** Acute kidney injury, Acute kidney disease, Progression to chronic kidney disease, Renal recovery, Renal scintigraphy

## Abstract

**Introduction:**

Renal scintigraphy (RS) is occasionally performed to assess the risk of persistent renal failure (PRF) in patients with acute kidney disease (AKD). However, its diagnostic performance has never been assessed.

**Methods:**

We identified all patients with AKD for whom RS was performed in our institution between 2010 and 2017. PRF was defined as persistently low (< 33% of baseline) estimated glomerular filtration rates (eGFR), 1 year after RS. Nuclear medicine specialists reviewed RS data and rated, for each patient, the likelihood of PRF (“PRF score”). We evaluated the performance to predict PRF (area under the ROC curve (AUC)) of RS-derived parameters such as renal accumulation index, accumulation slope, and new parameters derived from serial kidney activity counts. We tested the ability of those parameters to improve a clinical model including hypertension, diabetes, AKI severity and baseline eGFR. Finally, we conducted sensitivity analyses using alternate PRF definitions.

**Results:**

Among 97 patients included, 57 (59%) fulfilled the criteria for PRF. The PRF score was able to predict PRF with an AUC of 0.63. Similarly, the accumulation index and accumulation slope respective AUCs were 0.64 and 0.63. None of these parameters were able to improve the performance of the clinical model. Among new parameters, the 3rd/2nd minute activity ratio and 3rd/2nd minute activity slope had fair diagnostic performance (AUC 0.72 and 0.74, respectively) and improved the performance of the clinical model. Results were confirmed in sensitivity analyses.

**Conclusion:**

Conventional renal scintigraphy can identify patients at high risk of PRF with a high specificity but a low sensitivity. New parameters, with comparable diagnostic abilities can be obtained within three minutes of injection.

**Graphical abstract:**

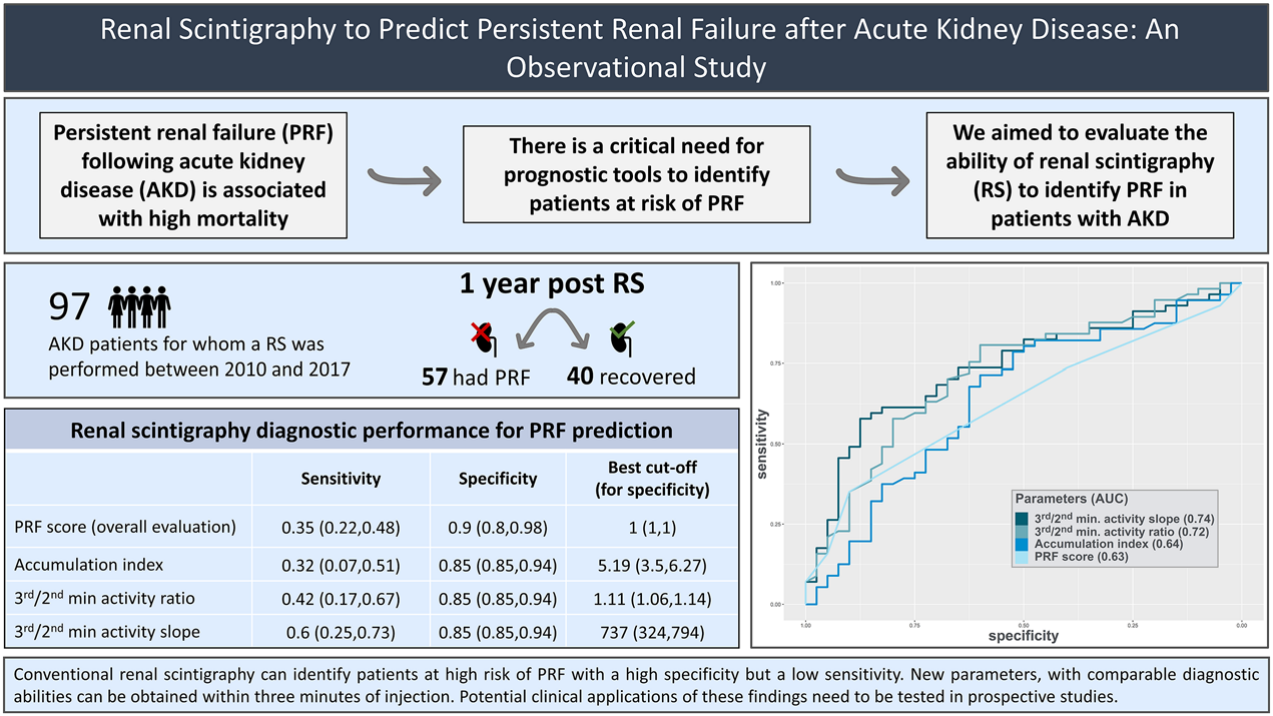

**Supplementary Information:**

The online version contains supplementary material available at 10.1007/s40620-023-01569-0.

## Introduction

Acute kidney injury (AKI) is diagnosed in 3–18% of adult patients admitted to the hospital [1]. In addition to its impact on short-term morbidity and mortality [2], AKI is associated with increased risk of chronic kidney disease (CKD) [3] and long-term dialysis dependence [4]. Given the health care and cost implications of these complications, renal recovery from AKI has been identified as an important research area [5]. In particular, there is a critical need for prognostic tools to assess kidney potential for recovery and identify patients at risk of persistent renal failure (PRF). Such tools would facilitate patient selection for nephrology follow-up, enable clinicians to provide adequate recommendations to the patients and perhaps drive new therapeutic strategies in the future. AKI is considered resolved when renal function returns to baseline values within 3 months. The 3-month period following AKI is usually referred to as acute kidney disease (AKD) which then may lead to CKD in case of no recovery during that period [6].

Renal scintigraphy (RS) provides information on renal function based on renal clearance and urinary excretion of an injected radiolabeled agent. RS has a wide range of clinical applications such as suspected obstructive nephropathy [7, 8], evaluation of congenital disorders of the urinary tract [9, 10] and reno-vascular hypertension [7, 11]. RS might be able to predict PRF after AKI. In an animal model of gentamicin-induced nephropathy, dimercaptosuccinic acid renography was correlated with renal histopathological results, suggesting its potential for characterization of renal damage extent [12]. In humans, dynamic RS is commonly performed in early graft failure following renal transplantation. It was shown to predict short- and long-term graft survival [13–15]. 99mTc-DTPA RS carried out within two days post-transplant was found to be a sensitive predictor of delayed graft function and 1-year serum creatinine (SCr) elevation (> 132 µmol/l; 1.5 mg/dl) [15].

In our institution, dynamic renal scintigraphy is occasionally performed to identify patients at risk of PRF, typically those with severe AKD, when an evaluation of renal potential for recovery is thought to impact clinical decision making. However, to date, this indication has not been confirmed by clinical studies. Accordingly, we aimed to evaluate the diagnostic performance of dynamic RS for persistent renal failure after AKI. We tested overall data interpretation as well as conventional and new RS-derived parameters individually.

## Methods

### Study design

This is an observational study conducted in a tertiary teaching hospital located in Lausanne, Switzerland. We retrospectively identified all consecutive adult (aged > 18 years) patients for whom a dynamic renal scintigraphy was performed between January 1, 2010 and December 31, 2017. Among them, we identified those who fulfilled the criteria for AKI or AKD, (according to a simplified version of the Kidney Disease Improving Global Outcomes (KDIGO) guidelines, see below) at the time of the examination [6, 16]. Patients with evidence or suspicion of obstructive nephropathy and those who had received a kidney transplant were excluded. For patients who underwent multiple RSs, only the first study was considered.

The study protocol was approved by the Ethics Committee Vaud (CER-VD 2019-00045). Given the observational nature of the study, the need for informed consent was waived and authorization was granted to use anonymized data unless patients had expressed their refusal for data reutilization.

### Definitions

#### Renal function

For all patients included in the study, we collected all the sCr levels measured in our institution in the 365 days before and after (± 90 days) RS. Renal function was estimated based on sCr levels at different time points: “baseline”, “time of RS” and “1 year”. We considered the following sCr levels: "baseline”: lowest value measured in the 365 days prior to RS; “time of RS": latest value measured in the 14 days prior to RS; “1 year”: lowest value measured in the 365 (± 90 days) following RS. At each time point, based on considered sCr value, we calculated an estimated glomerular filtration rate (eGFR) using the Chronic Kidney Disease Epidemiology Collaboration (CKD-EPI) formula assuming white race as default [6, 17]. At any time point, the need for renal replacement therapy was considered equivalent to an eGFR of 0 ml/min/1.73 m^2^.

According to the KDIGO definition, AKI was defined as a 50% increase of sCr at the time of RS compared to baseline value. Acute sCr elevation was confirmed by medical chart review either based on serial sCr measurements or on the mention in the medical documentation of an acute deterioration of renal function. Similarly, AKI stage was determined based on the relative increase of sCr relative to baseline value (Stage 1: 1.5–1.9-fold increase or ≥ 26.5 µmol/l increase; stage 2: 2.0–2.9-fold increase and stage 3: ≥ threefold increase or ≥ 353.6 µmol/l increase or renal replacement therapy (RRT) initiation) [6]. Patients with a significant sCr increase at the time of RS lasting > 90 days were not considered as AKI.

#### Persistent renal failure: percentage GFR recovery

In the absence of a consensus definition, we considered that a patient had persistent renal failure if index eGFR recovery was < 33%. Index GFR recovery was calculated in the following manner. We first estimated eGFR loss at the time of AKI:

$${\text{eGFR}}_{{{\text{loss}}}} = {\text{eGFR}}_{{{\text{baseline}}}} - {\text{eGFR}}_{{{\text{scinti}}}}$$

where eGFR_baseline_ stands for baseline eGFR and eGFR_scinti_ for eGFR estimated at the time of RS. We then calculated eGFR recovery:

$${\text{eGFR}}_{{{\text{recovery}}}} = {\text{eGFR}}_{{{1}\;{\text{year}}}} - {\text{eGFR}}_{{{\text{scinti}}}}$$

where e*GFR*_*1 year*_ stands for GFR estimated at the “1 year” time point. We were then able to compute the index eGFR recovery:

$${\text{index eGFR recovery}} = {\text{eGFR}}_{{{\text{recovery}}}} /{\text{eGFR}}_{{{\text{loss}}}} \times 100$$

**Fig. 1:**
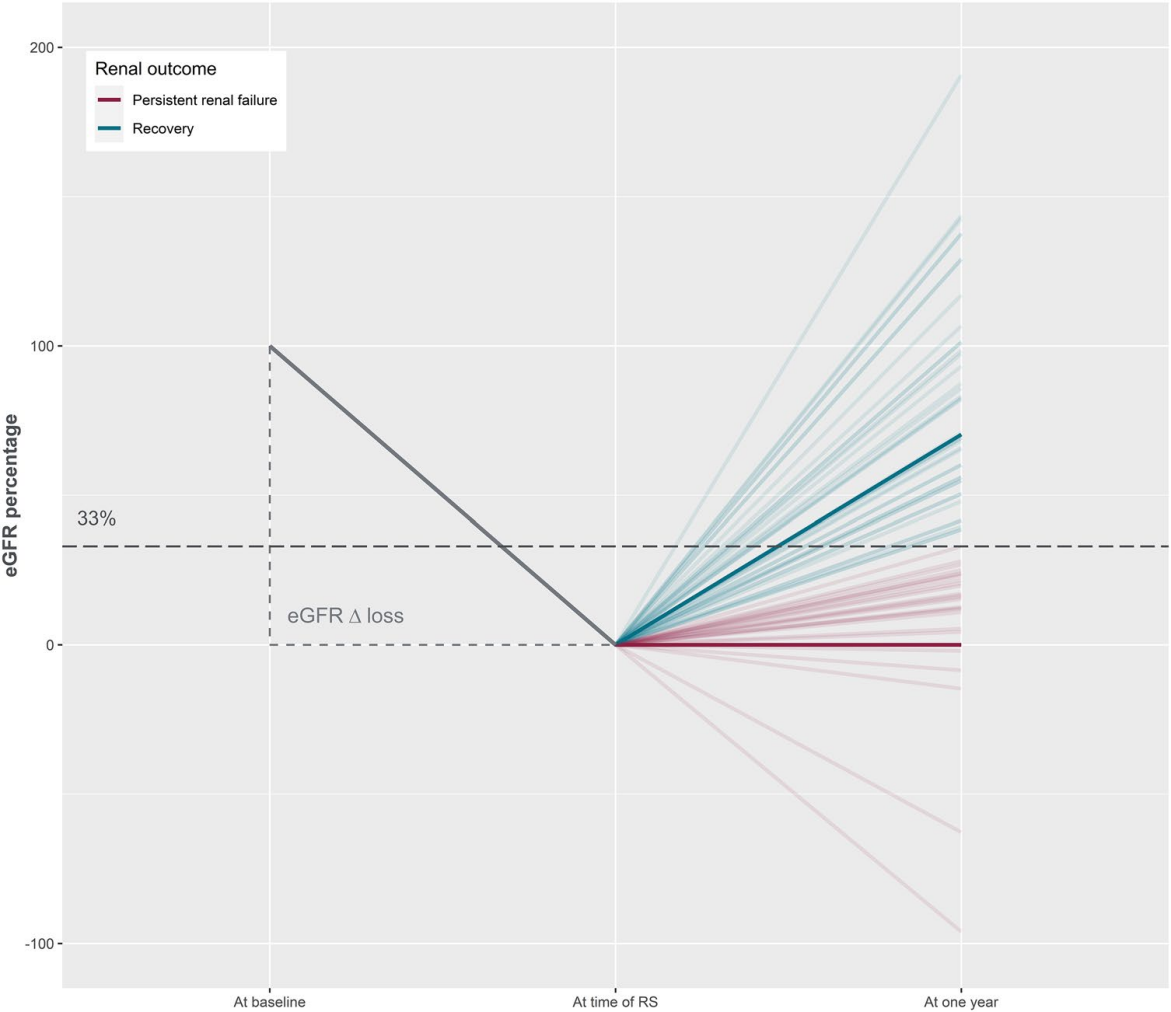
eGFR variation over time and persistent renal failure definition. Estimated glomerular filtration rate (eGFR) variation from baseline over time. The eGFR Δ loss describes the renal function loss from baseline to the time of acute kidney injury. We considered that patients had persistent renal failure (in red) if the eGFR recovery from AKI to follow-up was < 33% of the eGFR Δ loss. Patients with recovery > 33% of the eGFR Δ loss were classified in the recovery group (in blue). “Baseline” eGFR was calculated based on lowest available serum creatinine (sCR) value within 365 days prior to renal scintigraphy (RS). “1 year” eGFR was calculated based on lowest available sCr in the 425 days post RS. The need for renal replacement therapy was considered equivalent to an eGFR of 0 ml/min/1.73 m^2^. For the eGFR “at time of RS", we considered the latest sCr value measured prior to RS (< 14 days before RS)

An arbitrary cut off of 33% was chosen to define persistent renal failure. According to the percentage recovery calculation, patients were categorized into two categories: "recovery" or "persistent renal failure" (Fig. 1).

#### Sensitivity analyses

For confirmatory purposes, we applied alternate definitions of PRF. Alternate definitions were: (1) Percentage recovery < 25%; (2) Presence of severe CKD (GFR < 30 ml/min/1.73 m^2^) on follow-up, irrespective of baseline function; and (3) Follow-up eGFR < 50% of baseline eGFR, irrespective of AKI severity. For all definitions, we considered RRT dependence at follow-up as PRF. We also repeated our analyses in the subgroup of patients who were receiving RRT at the time of RS. In those patients, PRF was defined as ongoing need for RRT at the time of follow-up.

### Dynamic RS

Dynamic RSs were performed on a single head gamma camera (Millennium, GE Healthcare, Waukesha, WI, USA), with a posterior view dynamic acquisition using a 128 × 128 matrix (30 × 1 s frame followed by 117 × 10 s frame) starting immediately after the injection of 0.5 MBq/kg of Tc-99 m-MAG3 or iode-123-hippuran. Post-treatment of images was conducted on Xeleris (GE). The following parameters were considered and collected from the RS reports: relative function distribution (left vs. right) determined by the distribution of the relative activity in the two kidneys within one minute beginning at 30–50 s post-injection, time to maximal renal uptake (peak time), tracer elimination half-life, accumulation index (percentage of total injected activity excreted by the kidney within one minute), elimination index (ratio of the renal activity at peak time (max 3 min) to the renal activity at 20 min) and accumulation slope (slope of the time activity curve during one minute beginning at 30–50 s post-injection). The sum of the accumulation index and accumulation slope of the two kidneys was used in further statistical analysis. For the elimination index, the values of the best kidney were considered for further analysis.

Based on the images, reported RS parameters and time activity curves, the risk of PRF was estimated by two nuclear medicine specialists (MJ and MNL) blinded to all outcomes. They used a pre-established scale (1: low risk, 2: moderate risk, 3: high risk, 4: very high risk). The scores of the two readers were combined to form a mean PRF score which was dichotomized at 3 (high estimated PRF risk).

In a second stage, raw RS data were re-analyzed by one nuclear medicine specialist (MNL). Serial 1-min renal absolute activity counts of the best kidney during the 2nd, 3rd, 4th, 6th and 20th minutes post-injection were extracted. New RS parameters were computed for both kidneys: renal activity ratios and activity slopes between each time point.

### Statistical analyses

Data with normal distribution are reported as mean (SD) and compared using Student’s *t* test, while data with non-normal distribution are reported as median (interquartile range [IQR]) and compared using Mann–Whitney *U* test. Ordinal data are reported as number (percentage) and compared by means of Fisher’s exact or chi-square test as appropriate.

Multivariate logistic regression analyses were conducted for further investigations. Variables selected for inclusion in regression models were those with plausible rationale (age, hypertension, diabetes, AKI severity) and/or an alpha value of 0.1 or less in bivariate analyses. Odds ratios are presented with 95% confidence intervals.

For each parameter we also computed sensitivity, specificity (with binomial 95% confidence intervals), positive predictive value (PPV), negative predictive value (NPV) (with bootstrap 95% confidence intervals) as well as the area under the ROC curve (AUC). Cut-off points were selected with specificity above 0.85.

Inter-observer agreement was assessed with weighted Cohen kappa coefficient (κ).

For all analyses, we considered an alpha value of less than 0.05 to be statistically significant. Missing data are reported in supplementary table S1.

Statistical analyses were performed with R, version 3.6.2 (including extension packages dplyr, data.table, tableone, cutpointr, pROC, ggplot2).

## Results

### Patients’ demographics and group allocation

During the study period, 1938 RSs were performed in 1625 patients. Among them, we excluded 1835 studies for the following reasons: no evidence of AKI at time of RS (731), evidence of obstructive nephropathy (794), prior kidney transplantation (256), declined consent for data reutilization (43) and repeat examinations in a previously included patient (11). Hence, 103 RSs in 103 patients were included in the present study. Six patients with either no baseline or follow-up sCr could only be included in some sensitivity analyses.

At the time of RS, 91 patients (88.3%) were inpatients while 12 (11.7%) were outpatients. Admission was medical for 70 patients (68.0%), cardio-surgical for 22 (21.4%) and surgical for 11 (10.7%). Median time between AKI/AKD onset and RS was 14 days (IQR 22.7). Among patients with full dataset (97), 57 (58.8%) had not recovered > 33% from their eGFR loss 1 year after RS and were classified into the PRF group. The other 40 (41.2%) were classified into the “recovery” group.

As shown in Table 1, baseline characteristics were relatively well-balanced between the two groups, except for a higher percentage of patients with stage 3 AKI (62.5 versus 86%, *p* = 0.02) or requiring RRT (56.1% vs 42.5%, *p* = 0.3) in the PRF group.

**Table 1:** Patients' characteristics at baseline, at time of RS and 1-year outcomes

	Recovery (*N* = 40)	PRF (*N* = 57)	*p* value
Parameters at the time of RS			
Demographics			
Mean age, years (SD)	61.5 (13.9)	63.1 (15.8)	0.6
Gender, male, *n* (%)	30 (75.0)	40 (70.2)	0.8
Mean body weight, kg (SD)	76.3 (15.7)	76.8 (15.7)	0.9
Co-existing conditions, *n* (%)			
Chronic hypertension^a^	29 (74.4)	47 (83.9)	0.4
Diabetes mellitus^a^	10 (25.6)	17 (30.4)	0.8
Baseline renal function			
Number of kidney, one, *n* (%)	6 (15.0)	7 (12.3)	0.9
Median baseline creatinine, µmol/l (IQR)^b^	94.5 (66.2)	120.0 (78)	0.4
Median baseline clearance CKD-EPI, ml/min (IQR)^b^	64.5 (44.4)	50.1 (52.4)	0.3
Chronic kidney stage (KDIGO)^b^, *n* (%)			0.18
1	10 (25.0)	13 (25.5)	
2	12 (30.0)	6 (11.8)	
3	15 (37.5)	26 (51.0)	
4	3 (7.5)	6 (11.8)	
AKI/AKD characteristics			
Median time between AKI onset and RS, days (IQR)^c^	11.5 (20.7)	14 (25)	0.235
Type of patient, *n* (%)			0.501
Medical	25 (62.5)	42 (73.7)	
Surgical	4 (10)	4 (7)	
Cardio-surgical	11 (27.5)	11 (19.3)	
Sepsis or septic shock, yes, n (%)	2 (5)	9 (15.8)	0.240
Renal function at RS			
Median creatinine, µmol/L (IQR)^****^	277.0 (123)	369.0 (125)	0.002
Acute kidney injury stage (KDIGO), n (%)			0.016
1	6 (15.0)	5 (8.8)	
2	9 (22.5)	3 (5.3)	
3	25 (62.5)	49 (86.0)	
On RRT, *n* (%)	17 (42.5)	32 (56.1)	0.3
Outcomes 1-year post RS			
Creatinine, µmol/l, median (IQR)^d^	126.5 (57.2)	262.0 (77.5)	
Clearance, ml/min/1.73 m^2^, (CKD-EPI), median (IQR)^d^	47.6 (26.5)	21.1 (9.7)	
Ongoing RRT, *n* (%)	0 (0.0)	34 (59.6)	
Death, *n* (%)	5 (12.5)	17 (29.8)	

*RS* renal scintigraphy, *SD* standard deviation, *IQR* interquartile range, *GFR* glomerular filtration rate, *RRT* renal replacement therapy, *KDIGO* Kidney Disease: Improving Global Outcomes, *CKD-EPI* Chronic Kidney Disease Epidemiology Collaboration

^a^Missing for 2 patients

^b^Missing for 6 patients

^c^Missing for 1 patient

^d^Patients on RRT not included

### Clinical outcomes

During the follow-up period, 22 patients (22.7%) died: 5 (12.5%) in the recovery group and 17 (29.8%) in the PRF group. Thirty-four patients (35%) were receiving RRT at the “1 year” time point (Table 1).

### Prognostic ability of RS parameters

Renal scintigraphy parameters are presented in Table 2. Examples of RS output are presented in Figure S2 (Supplemental Material).

**Table 2:** Renal scintigraphy parameters

	Recovery (*N* = 40)	PRF (*N* = 57)	*p* value
Calculated RS parameters, median (IQR)			
Accumulation index^a^	7.8 (4.6)	6.4 (2.8)	0.017
Elimination index^b^	0.9 (0.4)	0.9 (0.4)	0.9
Accumulation slope^c^	0.5 (0.4)	0.3 (0.3)	0.033
Calculated RS activity ratio, median (IQR)			
3rd/2nd minute activity ratio	1.17 (0.05)	1.12 (0.07)	< 0.001
4th/2nd minute activity ratio	1.26 (0.15)	1.20 (0.14)	0.001
6th/2nd minute activity ratio	1.40 (0.22)	1.26 (0.25)	0.001
Calculated RS activity slope, median (IQR)			
3rd/2nd minute activity slope	1089.5 (703.3)	663.0 (677)	< 0.001
4th/2nd minute activity slope	833.5 (568.3)	502.5 (481.5)	< 0.001
6th/2nd minute activity slope	619.6 (470.4)	303.8 (409.3)	< 0.001
Renal recovery score, *n* (%)			
Examiner 1			0.016
1	6 (15.0)	6 (10.5)	
2	21 (52.5)	17 (29.8)	
3	11 (27.5)	19 (33.3)	
4	2 (5.0)	15 (26.3)	
Examiner 2			0.16
1	14 (35.0)	21 (36.8)	
2	21 (52.5)	19 (33.3)	
3	4 (10.0)	12 (21.1)	
4	1 (2.5)	5 (8.8)	
Mean PRF score ≥ 3	4 (10.0)	20 (35.1)	0.008

The sum of the accumulation index and accumulation slope of the two kidneys was considered in this analysis. For the other parameters, the values of the best kidney were considered

*IQR* interquartile range, *RS* renal scintigraphy

^a^Missing for 1 patient

^b^Missing for 2 patients

^c^Missing for 3 patients

#### Nuclear medicine specialist interpretation

Overall agreement between the two evaluators was poor (34.0%, weighted kappa 0.23). Individual performance is depicted in Fig. S1. The proportion of patients with a mean PRF score ≥ 3 was higher in the PRF group than in the recovery group (*p* = 0.008). However, as shown in Table 3, the association disappeared after correction for age, hypertension, diabetes, baseline sCr and AKI severity (OR 3.4, CI 1.04–13, *p* = 0.057). A PRF score of ≥ 3 could predict PRD with 90% specificity and 35% sensitivity (PPV 83%, NPV 49%). The AUC for a mean PRF score ≥ 3 was 0.63 (CI 0.55–0.7) (Table 4, Fig. 3).

**Table 3:** Multivariable analyses for Persistent renal failure prediction

	2.5–97.5% CI				
	*n* patients	Odds ratio	Lower	Upper	*p* value
Standard model					
+ Accumulation index	88	0.90	0.77	1.05	0.18
+ Elimination index	87	1.62	0.34	8.11	0.5
+ Accumulation slope	86	0.31	0.05	1.28	0.14
+ Mean PRF score ≥ 3	89	3.4	1.04	13.6	0.057
+ 3rd/2nd minute activity ratio	89	6.4 × 10^–5^	9.4 × 10^–9^	0.104	0.02
+ 3rd/2nd minute activity slope	89	0.999	0.998	1	0.019

The relationship between renal scintigraphy parameters and persistent renal failure was explored in a multivariate logistic regression model. Variables included in the standard model were age, presence of hypertension, presence of type 2 diabetes, acute kidney injury stage according to the KDIGO guidelines and baseline glomerular filtration rate according to the Chronic Kidney Disease Epidemiology Collaboration (CKD-EPI) equation. The sum of the accumulation index and accumulation slope of the two kidneys was considered in this analysis. For the other parameters, the values of the best kidney were considered

*PRF* persistent renal failure

**Table 4:** Diagnostic performance of Clinical score and RS-derived parameters

	Area under curve	Best cut-off (for specificity)	Sensitivity	Specificity	PPV	NPV
Conventional RS parameters, median (IQR)						
Accumulation index^a^	0.64 (0.53,0.76)	5.19 (3.5,6.27)	0.32 (0.07,0.51)	0.85 (0.85,0.94)	0.75 (0.45,0.89)	0.47 (0.35,0.6)
Elimination index^b^	0.51 (0.39,0.62)	1.35 (1.08,1.55)	0.11 (0.02,0.38)	0.9 (0.85,0.97)	0.6 (0.25,0.85)	0.41 (0.32,0.52)
Accumulation slope^c^	0.63 (0.51,0.74)	0.2 (0.08,0.38)	0.2 (0.06,0.59)	0.85 (0.85,0.95)	0.65 (0.44,0.89)	0.44 (0.35,0.62)
Calculated RS activity ratio, median (IQR)						
3rd/2nd minute activity ratio	0.72 (0.62,0.82)	1.11 (1.06,1.14)	0.42 (0.17,0.67)	0.85 (0.85,0.94)	0.8 (0.67,0.92)	0.51 (0.37,0.67)
4th/2nd minute activity ratio	0.69 (0.58,0.8)	1.16 (1.08,1.21)	0.39 (0.18,0.61)	0.85 (0.85,0.94)	0.79 (0.67,0.91)	0.49 (0.37,0.64)
6th/2nd minute activity ratio	0.69 (0.58,0.79)	1.22 (1.11,1.28)	0.42 (0.21,0.6)	0.88 (0.85,0.97)	0.83 (0.75,0.94)	0.51 (0.4,0.64)
Calculated RS activity slope, median (IQR)						
3rd/2nd minute activity slope	0.74 (0.63,0.83)	737 (324,794)	0.6 (0.25,0.73)	0.85 (0.85,0.94)	0.85 (0.76,0.94)	0.6 (0.41,0.72)
4th/2nd minute activity slope	0.72 (0.62,0.82)	572.5 (377,601.5)	0.56 (0.34,0.67)	0.85 (0.85,0.95)	0.84 (0.77,0.94)	0.58 (0.44,0.69)
6th/2nd minute activity slope	0.71 (0.61,0.82)	286.25 (148.5,448.5)	0.49 (0.26,0.65)	0.85 (0.85,0.95)	0.82 (0.74,0.93)	0.54 (0.41,0.67)
Mean PRF score ≥ 3	0.63 (0.55,0.7)	1 (1,1)	0.35 (0.22,0.48)	0.9 (0.8,0.98)	0.83 (0.68,0.96)	0.49 (0.39,0.6)

Table of computed sensitivity, specificity, area under curve (with binomial 95% confidence intervals), positive predictive value and negative predictive value (with bootstrap 95% confidence intervals). Cut-off points were performed with fixed specificity above 0.85. The sum of the accumulation index and accumulation slope of the two kidneys was considered in this analysis. For the other parameters, the values of the best kidney were considered

*IQR* interquartile range, *PPV* positive predictive value, *NPV* negative predictive value, *RS* renal scintigraphy, *PRF* persistent renal failure

^a^Missing for 1 patient

^b^Missing for 2 patients

^c^Missing for 3 patients

#### Individual conventional RS parameters

**Fig. 2:**
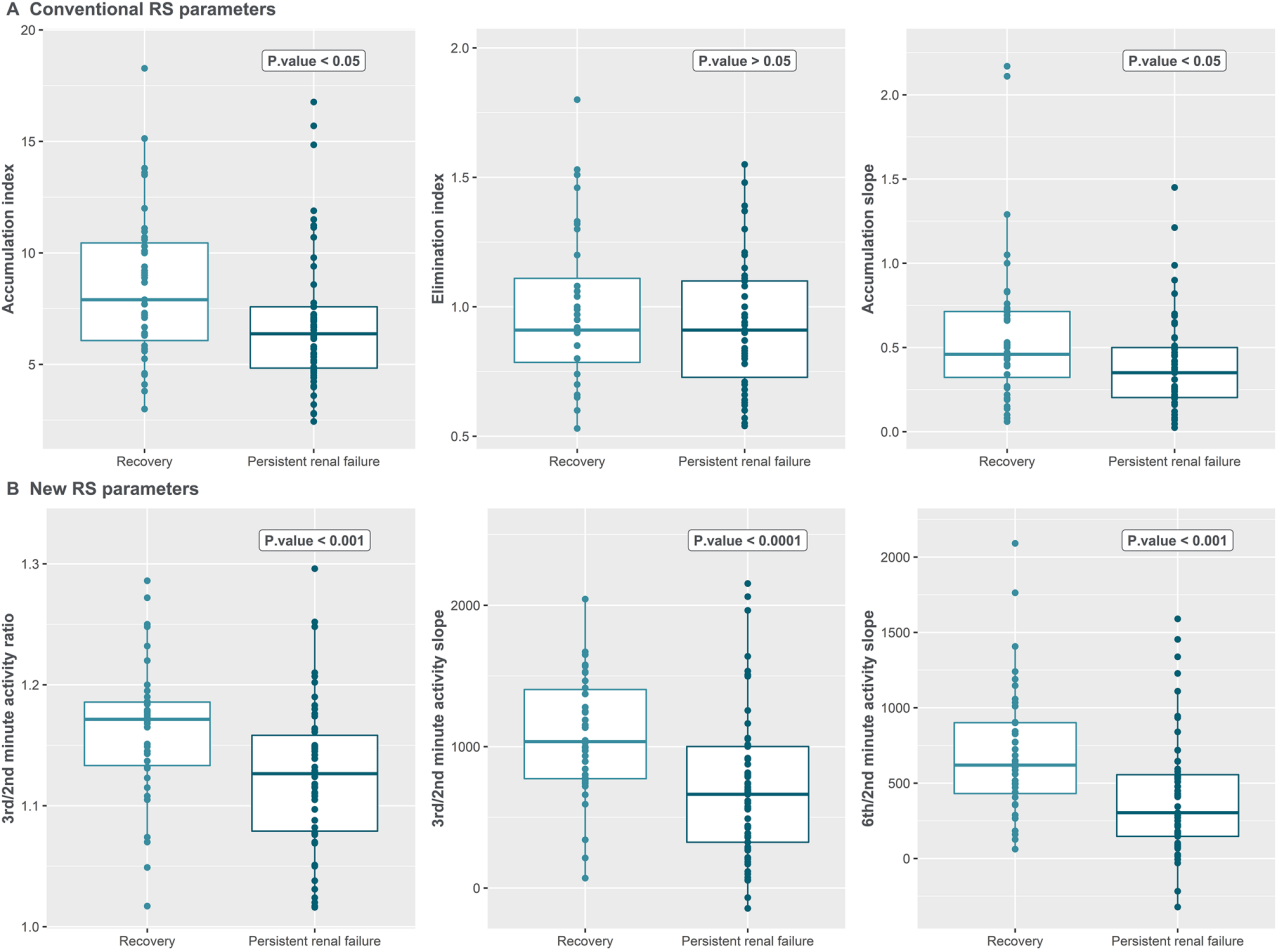
Renal scintigraphy parameters comparison by box-and-whiskers plot between renal recovery and persistent renal failure. *Min.* minute, *RS* renal scintigraphy. The lower and upper hinges correspond to the first and third quartiles. The middle hinge corresponds to the median. The upper whisker and lower whisker, respectively, extends from the upper and lower hinge to the largest and lowest value no further than 1.5 times the interquartile range. The sum of the accumulation index and accumulation slope of the two kidneys was considered in this analysis. For the other parameters, the values of the best kidney were considered. Outcome was missing for 6 patients (No creatinine available at baseline or in the follow-up period). Missing values for total accumulation index (1 patient), elimination index (2 patients), accumulation slope (3 patients)

As shown in Fig. 2a, accumulation index and accumulation slope were both higher in the recovery group compared with the PRF group (resp. 7.8 vs 6.4, *p* = 0.017 and 0.5 vs 0.3, *p* = 0.033). However, this difference disappeared after correction for age, hypertension, diabetes, baseline sCr and AKI severity (resp. OR 0.90, CI 0.77–1.05, *p* = 0.18 and OR 0.31, CI 0.05–1.28, *p* = 0.14 see Table 3).

The three parameters (Table 4) had PPV ranging between 60 and 75% and NPV between 41 and 47%. Their AUC ranged between 0.51 (elimination index) and 0.64 (accumulation index).

#### New RS parameters

Among all renal activity ratios and slopes tested, those related to the second minute (3/2, 4/2, etc.) appeared to have the strongest differentiating capacity between the two groups and were considered for further analyses.

As shown in Fig. 2b, the ratio of the renal activity count in the second and the third minute (3/2 ratio) as well as the calculated slope between these two data points (3/2 slope) were both higher in the recovery group compared to the PRF group (resp. 1.17 vs 1.12, and 1089 vs 663, both *p* < 0.001). These associations remained after correction for age, hypertension, diabetes, baseline sCr and AKI severity for both the 3/2 ratio (OR 6.4 × 10^–5^, CI 9.4 × 10^–9^–0.11, *p* = 0.02) and the 3/2 slope (OR 0.999, CI 0.998–1.0, *p* = 0.019).

**Fig. 3:**
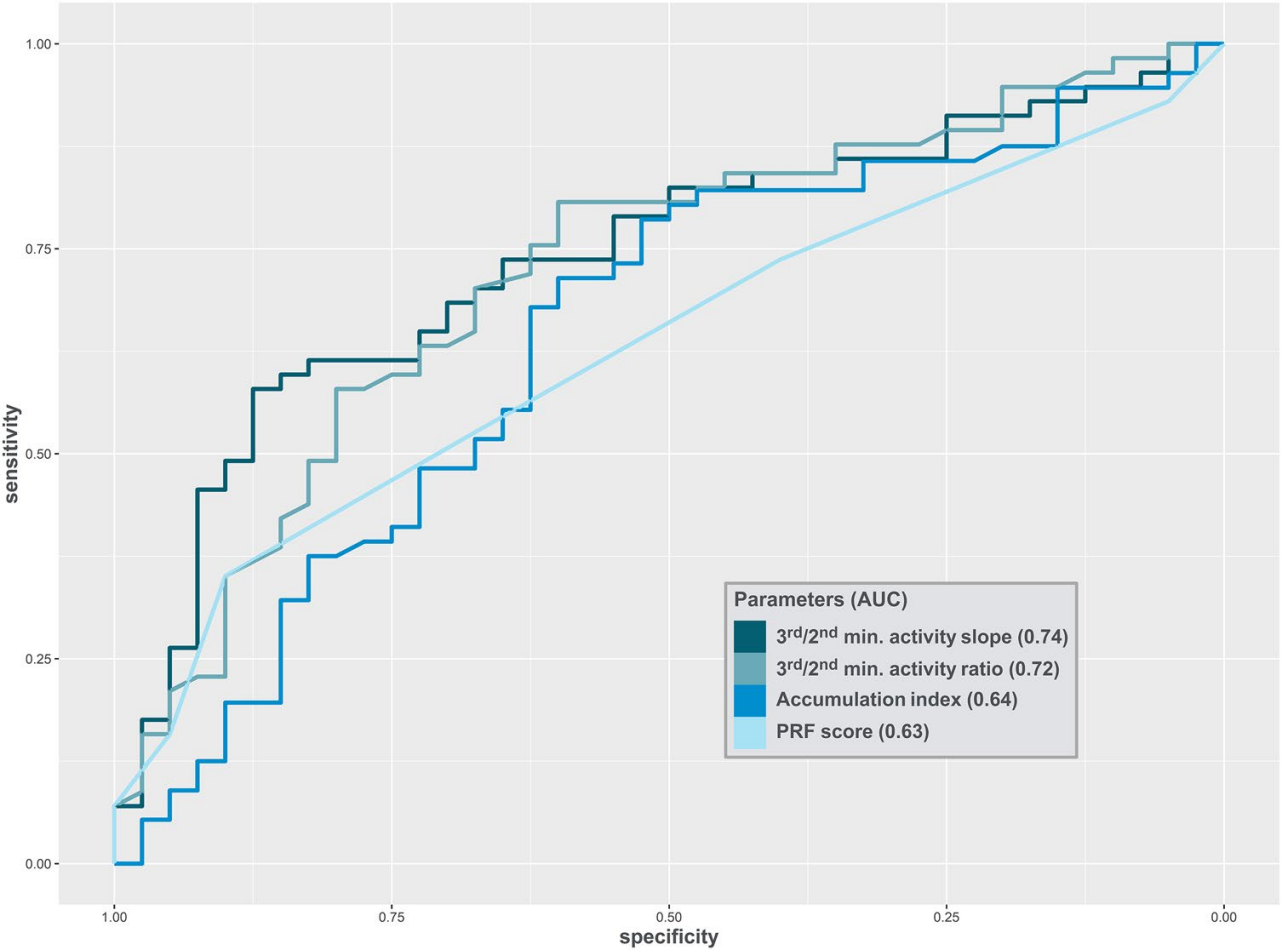
Receiver operating characteristic (ROC) curves depicting clinical score and RS-derived parameters diagnostic performance for PRF. *AUC* area under curve, *min.* minute, *PRF* persistent renal failure. The sum of the accumulation index of the two kidneys was considered in these analysis. For the 3rd/2nd minute activity ratio and slope, values of the best kidney were considered. Outcome was missing for 6 patients (no creatinine available at baseline or in the follow-up period). Total accumulation index was missing for 1 patient

Best cut-offs for specificity were 1.11 and 737. With those cut-offs, the 3/2 ratio was able to predict PRF with 85% specificity and 42% sensitivity (PPV 80%, NPV 51%) and the 3/2 slope with 85% specificity and 60% sensitivity (PPV 82% NPV 49%). The respective AUCs were 0.72 (CI 0.62–0.82) and 0.74 (CI 0.63–0.83) (Table 4, Fig. 3). Both parameters performed better than conventional RS parameters and nuclear medicine specialist overall interpretation.

### Sensitivity analyses

All analyses were repeated using three alternate definitions of persistent AKI and in the RRT subgroup. Detailed results are presented in the Supplemental material (table S2-S4 and S6-S9). Overall, these analyses confirm our findings. In particular, the PRF score performance was poor. It was associated with PRF after correction for comorbidities only in alternative definition 2 (*p* = 0.024) with an AUC of 0.62 (CI 0.5–0.73). Diagnostic performance of conventional RS parameters was similar in terms of specificity, sensitivity and AUC. None was associated with PRF after correction for comorbidities in all definitions. Finally, the performance of the 3/2 ratio and 3/2 slope remained similar with higher AUC than PRF score and conventional parameters for all alternative definitions. The 3/2 slope remained a predictor after correction for comorbidities with alternative definitions 1 and 2, but not with alternative definition 3. Similar results were also obtained when analyses were restricted to patients on RRT at the time of RS. In these patients, RS had low sensitivity and high specificity for predicting PRF (supplemental material table S5 and S10).

## Discussion

### Key findings

We conducted an observational study aiming to evaluate the diagnostic performance of RS to predict persistent renal failure in patients with AKI. We evaluated 103 consecutive RS examinations in 103 patients with AKI. We found that overall assessment as well as individual parameters such as accumulation index and accumulation slope were associated with PRF. However, no association remained after correction for age, hypertension, diabetes, baseline sCr and AKI severity. We found that these parameters could predict PRF with a high specificity, but a low sensitivity. On the other hand, we tested new indices, calculated based on renal counts in the first three minutes. These parameters were strongly associated with PRF and with higher AUC than conventional parameters, and their association with outcomes remained after correction for comorbidities.

### Comparison with previous studies

To the best of our knowledge, all previous studies aiming to evaluate RS ability to predict kidney recovery only included patients with kidney transplants [7, 13]. In this indication, several parameters appear to be associated with early and late kidney graft survival. Similar to the 3/2 ratio and 3/2 slope, the authors examined the tubular function slope (linear fit of the curve between 50 and 110 s post tracer injection), the corrected tubular extraction rate ([cTER], first to second minute renal uptake rate corrected for body surface) and the average upslope (the slope between counts at 20 s and 3 min). These three parameters focus on the extraction/uptake phase occurring in the first minutes of RS. Tubular function slope was shown to be lower in patients with delayed graft function and to be a predictor of graft failure at 1 year with an area under curve of 0.70 [18, 19]. Corrected TER and the average upslope had high specificity and sensitivity to detect delayed graft function one week after transplant [20, 21].

The R20/3 parameter (renal count ratio at 20 and 3 min), was associated with graft survival and function at 1 year in previous studies [13, 15, 22]. We did not observe such an association. This might be related to the severely impaired renal excretion capacity observed in patients with AKI. Indeed, tracer excretion phase was severely affected in most patients in our study.

Other techniques to predict renal recovery after AKI have been assessed. The main ones include renal resistive index by US Doppler and biomarkers such as neutrophil gelatinase-associated lipocalin (NGAL) and cystatin C. Despite promising previous results [23, 24], renal resistive index was recently shown to be a poor predictor of persistent AKI with AUC close to 0.58 in a large multicenter study [25]. However, these studies were more focused on short term (48–72 h) recovery. Urinary NGAL was found to be a predictor of RRT weaning and patient survival at 60 days after AKI with an AUC of 0.66 [26]. When associated with other biomarkers and with a clinical model, the AUC increased up to 0.93. However, these findings need to be confirmed in larger studies as NGAL has been shown to be largely non-specific [27–33].

### Strengths and limitations

This study has several strengths. it is the first to evaluate the ability of RS to predict PRF in patients with AKI or AKD. All 103 RSs were reviewed by two nuclear medicine specialists blinded to the outcome. Additional parameters, based on renal absolute counts, were calculated and extracted at five different time points. Our findings appear robust as they were confirmed by several sensitivity analyses. Our proposed new parameters have higher robustness compared to traditional parameters. Indeed, they rely on activity ratios and slopes which makes them independent of factors influencing measurements such as renal shape, background noise or attenuation correction method.

However, this study also has limitations worth discussing. First, it is a retrospective single-center study on a relatively small and heterogeneous group of patients. sCr levels were collected from our institution’s database whenever available and not at specific time points. Some measurements were obtained in the outpatient clinic and others in the hospital. SCr values may therefore not be indicative of true baseline or recovery values. However, the associated bias is likely to apply to patients in both groups. Second, in the absence of a consensus definition of renal recovery and persistent renal failure, we had to use a custom definition. This definition is debatable however, alternate definitions were tested in sensitivity analyses and did not alter our main findings. Third, we did not exclude patients who had died during the follow-up period. Such patients had less time to recover renal function. Finally, we did not use the full KDIGO criteria for AKI definition since we did not account for urinary output. Fourth, the timing of RS relative to AKI onset was not standardized and might have influenced our results. However, all patients had AKD at the time of RS.

### Clinical significance

Our data suggest that renal scintigraphy can identify patients with a very high likelihood of PRF. Such knowledge could enable to select patients who might benefit from nephrology follow-up. It could be informative for clinical decision-making in numerous clinical situations (for instance RRT initiation, choice of chemotherapy, palliative care initiation). Finally, in clinical research, it could be used to exclude patients with a very low chance of benefiting from a nephroprotective strategy.

On the other hand, the observed low sensitivity indicates the inability of the technique to predict renal *recovery*. Given the multiple clinical scenarios of recovery/relapse that might follow an examination, it is actually very unlikely that any test could reliably predict recovery. At best, a perfect test might determine a potential for recovery which may or may not be fulfilled according to future unpredictable clinical elements. For instance, a patient estimated to have a high potential for recovery might suffer from recurring kidney injuries such as sepsis, trauma or receive nephrotoxins and not fulfill his/her potential for recovery.

The new parameters proposed have other clinical significance. In this study, the interobserver agreement based on conventional RS interpretation was poor, revealing the need to integrate more robust parameters to evaluate PRF. Including these new parameters in the overall evaluation seems to be promising to improve interobserver agreement. In addition to improving RS interpretation, these new parameters might open doors to the application of nuclear medicine in the intensive care unit, reducing the risk of mobilization of those fragile patients. Indeed, techniques such as free-hand single photon emission tomography might be performed at the bedside [34]. This technique is however limited by the restricted field of view and acquisition protocols. Hence, a parameter harboring similar predictive capacities as the standard RS but only requiring three minutes of acquisition time could be of great clinical interest. This, of course needs to be confirmed in prospective studies.

### Conclusions

Conventional renal scintigraphy can identify patients at high risk of persistent renal failure with a high specificity but a low sensitivity. New parameters, with comparable diagnostic abilities can be obtained within three minutes of injection. Potential clinical applications of these findings need to be tested in prospective studies.

## Supplementary Information

Below is the link to the electronic supplementary material.

Supplementary file1 (DOCX 1015 KB)

## Data Availability

The datasets used and/or analysed during the current study are available from the corresponding author on reasonable request.
