# Supplementary material for: Renal scintigraphy to predict persistent renal failure after acute kidney injury: an observational study
Source: J Nephrol. 2023 Feb 2;36(4):1047–58. doi: 10.1007/s40620-023-01569-0 (PMC10226915; doi:10.1007/s40620-023-01569-0)
Supplement: Supplementary file 1 — Supplementary file1 (DOCX 1015 KB) [file 40620_2023_1569_MOESM1_ESM.docx]

**Supplemental Material to “Renal Scintigraphy to Predict Persistent Renal Failure after Acute Kidney Injury: An Observational Study”**

| **Patients (N = 103)** |  |
| --- | --- |
|  | **Missing values, n (%)** |
| **Patients' demographics and general characteristics** |  |
| **Renal function parameters** |  |
| Baseline Creatinine | 10 (9.7) |
| Baseline clearance | 10 (9.7) |
| Chronic kidney stage (KDIGO) | 10 (9.7) |
| **Co-existing conditions** |  |
| Chronic hypertension | 5 (4.8) |
| Diabetes mellitus | 5 (4.8) |
| **RS characteristics and parameters** |  |
| Accumulation index | 1 (1.0) |
| Elimination index | 2 (1.9) |
| Accumulation slope | 3 (2.9) |
| **Renal outcomes** |  |
| Creatinine or RRT status | 5 (4.8) |
| Chronic kidney stage (KDIGO) | 5 (4.8) |
| **Persistent renal failure definitions** |  |
| Main definition | 6 (5.8) |
| Alternative definition 1 | 6 (5.8) |
| Alternative definition 2 | 5 (4.8) |
| Alternative definition 3 | 6 (5.8) |
| RRT subgroup | 4 (3.9) |

**Table S1. Missing values for patient’s demographics, RS parameters and outcome**

RRT, renal replacement therapy; KDIGO, Kidney Disease: Improving Global Outcomes; RS, renal scintigraphy

|  | **Recovery**  **(N=44)** | **PRF (N=53)** | **P value** |
| --- | --- | --- | --- |
| **Deceased before day 425, n (%)** | 7 (15.9) | 15 (28.3) |  |
| **Renal outcomes** |  |  |  |
| Creatinine, µmol/L, median (IQR) ^*^ | 131.5 (65.7) | 262.0 (64.5) |  |
| Clearance, mL/min/1.73 m^2^, (CKD-EPI), median (IQR) ^*^ | 44.0 (28.4) | 21.1 (6.2) |  |
| Current RRT, n (%) | 0 (0.0) | 34 (64.2) |  |
| **Calculated RS parameters, median (IQR)** |  |  |  |
| Accumulation index^**^ | 7.3 (4.3) | 6.4 (2.8) | 0.05 |
| Elimination index^***^ | 0.9 (0.4) | 0.9 (0.4) | 0.7 |
| Accumulation slope^****^ | 0.4 (0.4) | 0.3 (0.3) | 0.045 |
| **Calculated RS activity ratio, median (IQR)** |  |  |  |
| 3^rd^/2^nd^ minute activity ratio | 1.17 (0.06) | 1.12 (0.07) | 0.001 |
| 4^th^/2^nd^ minute activity ratio | 1.26 (0.16) | 1.20 (0.14) | 0.005 |
| 6^th^/2^nd^ minute activity ratio | 1.37 (0.23) | 1.26 (0.25) | 0.005 |
| **Calculated RS activity slope, median (IQR)** |  |  |  |
| 3^rd^/2^nd^ minute activity slope | 1036.0 (705.6) | 663.0 (560) | < 0.001 |
| 4^th^/2^nd^ minute activity slope | 795.0 (575.9) | 502.5 (424.5) | < 0.001 |
| 6^th^/2^nd^ minute activity slope | 608.5 (506.4) | 303.8 (409.3) | 0.001 |
| **Renal recovery score, n (%)** |  |  |  |
| Examiner 1 |  |  | 0.17 |
| 1 | 6 (13.6) | 6 (11.3) |  |
| 2 | 21 (47.7) | 17 (32.1) |  |
| 3 | 13 (29.5) | 17 (32.1) |  |
| 4 | 4 (9.1) | 13 (24.5) |  |
| Examiner 2 |  |  | 0.2 |
| 1 | 16 (36.4) | 19 (35.8) |  |
| 2 | 22 (50.0) | 18 (34.0) |  |
| 3 | 5 (11.4) | 11 (20.8) |  |
| 4 | 1 (2.3) | 5 (9.4) |  |
| Mean PRF score ≥ 3 | 5 (11.4) | 19 (35.8) | 0.009 |

**Table S2 - General outcomes – Alternative definition 1**

SD, standard deviation; IQR, interquartile range; GFR, glomerular filtration rate; RRT, renal replacement therapy; KDIGO, Kidney Disease: Improving Global Outcomes; CKD-EPI, Chronic Kidney Disease Epidemiology Collaboration, RS, renal scintigraphy; PRF, persistent renal failure.

Persistent renal failure defined as <25% recovery in estimated GFR within 425 days of RS or as the need for renal replacement therapy. The sum of the accumulation index and accumulation slope of the two kidneys was considered in these analysis. For the other parameters, the values of the best kidney were considered.

^*^ patients on RRT not included

^**^ missing for 1 patient ^***^ missing for 2 patients ^****^ missing for 3 patients

|  | **Recovery**  **(N=40)** | **PRF (N=58)** | **P value** |
| --- | --- | --- | --- |
| **Deceased before day 425, n (%)** | 4 (10.0) | 18 (31.0) |  |
| **Renal outcomes** |  |  |  |
| Creatinine, µmol/L, median (IQR) ^*^ | 116.5 (53.3) | 265.0 (70.5) |  |
| Clearance, mL/min/1.73 m^2^, (CKD-EPI), median (IQR) ^*^ | 49.2 (32.2) | 21.0 (7.7) |  |
| Current RRT, n (%) | 0 (0.0) | 34 (58.6) |  |
| **Calculated RS parameters, median (IQR)** |  |  |  |
| Accumulation index^**^ | 8.3 (4.5) | 6.4 (2.8) | 0.011 |
| Elimination index^***^ | 0.9 (0.4) | 0.9 (0.4) | 0.7 |
| Accumulation slope^****^ | 0.5 (0.4) | 0.3 (0.3) | 0.01 |
| **Calculated RS activity ratio, median (IQR)** |  |  |  |
| 3^rd^/2^nd^ minute activity ratio | 1.17 (0.06) | 1.13 (0.08) | 0.002 |
| 4^th^/2^nd^ minute activity ratio | 1.25 (0.18) | 1.20 (0.14) | 0.017 |
| 6^th^/2^nd^ minute activity ratio | 1.42 (0.25) | 1.27 (0.17) | 0.003 |
| **Calculated RS activity slope, median (IQR)** |  |  |  |
| 3^rd^/2^nd^ minute activity slope | 1145.0 (699) | 682.0 (556) | < 0.001 |
| 4^th^/2^nd^ minute activity slope | 874.8 (759.7) | 526.2 (433.2) | < 0.001 |
| 6^th^/2^nd^ minute activity slope | 647.9 (522.6) | 324.0 (381.9) | < 0.001 |
| **Renal recovery score, n (%)** |  |  |  |
| Examiner 1 |  |  | 0.035 |
| 1 | 6 (15.0) | 6 (10.3) |  |
| 2 | 21 (52.5) | 17 (29.3) |  |
| 3 | 10 (25.0) | 21 (36.2) |  |
| 4 | 3 (7.5) | 14 (24.1) |  |
| Examiner 2 |  |  | 0.2 |
| 1 | 15 (37.5) | 20 (34.5) |  |
| 2 | 20 (50.0) | 20 (34.5) |  |
| 3 | 4 (10.0) | 13 (22.4) |  |
| 4 | 1 (2.5) | 5 (8.6) |  |
| Mean PRF score ≥ 3 | 4 (10.0) | 21 (36.2) | 0.004 |

**Table S3 - General outcomes – Alternative definition 2**

SD, standard deviation; IQR, interquartile range; GFR, glomerular filtration rate; RRT, renal replacement therapy; KDIGO, Kidney Disease: Improving Global Outcomes; CKD-EPI, Chronic Kidney Disease Epidemiology Collaboration, RS, renal scintigraphy; PRF, persistent renal failure.

Persistent renal failure defined as severe CKD (GFR < 30 mL/min/1.73m2) on follow-up, irrespective of baseline function. The sum of the accumulation index and accumulation slope of the two kidneys was considered in these analysis. For the other parameters, the values of the best kidney were considered.

^*^ patients on RRT not included ^**^ missing for 1 patient ^***^ missing for 2 patients ^****^ missing for 3 patients

|  | **Recovery (N=45)** | **PRF (N=52)** | **P value** |
| --- | --- | --- | --- |
| **Deceased before day 425, n (%)** | 9 (20.0) | 13 (25.0) |  |
| **Renal outcomes** |  |  |  |
| Creatinine, µmol/L, median (IQR) ^*^ | 130.0 (83) | 268.0 (88.5) |  |
| Clearance, mL/min/1.73 m^2^, (CKD-EPI), median (IQR) ^*^ | 44.3 (31.8) | 21.1 (11.5) |  |
| Current RRT, n (%) | 0 (0.0) | 34 (65.4) |  |
| **Calculated RS parameters, median (IQR)** |  |  |  |
| Accumulation index^**^ | 7.2 (4.4) | 6.5 (2.7) | 0.19 |
| Elimination index^***^ | 0.9 (0.4) | 0.9 (0.4) | 0.068 |
| Accumulation slope^****^ | 0.4 (0.4) | 0.3 (0.3) | 0.12 |
| **Calculated RS activity ratio, median (IQR)** |  |  |  |
| 3^rd^/2^nd^ minute activity ratio | 1.16 (0.06) | 1.12 (0.09) | 0.003 |
| 4^th^/2^nd^ minute activity ratio | 1.24 (0.16) | 1.20 (0.15) | 0.035 |
| 6^th^/2^nd^ minute activity ratio | 1.35 (0.23) | 1.28 (0.26) | 0.1 |
| **Calculated RS activity slope, median (IQR)** |  |  |  |
| 3^rd^/2^nd^ minute activity slope | 1014.0 (594) | 640.0 (688) | < 0.001 |
| 4^th^/2^nd^ minute activity slope | 742.0 (474) | 559.0 (560.9) | 0.006 |
| 6^th^/2^nd^ minute activity slope | 559.5 (539) | 419.6 (447.3) | 0.061 |
| **Renal recovery score, n (%)** |  |  |  |
| Examiner 1 |  |  | 0.2 |
| 1 | 6 (13.3) | 6 (11.5) |  |
| 2 | 20 (44.4) | 18 (34.6) |  |
| 3 | 15 (33.3) | 15 (28.8) |  |
| 4 | 4 (8.9) | 13 (25.0) |  |
| Examiner 2 |  |  | 0.2 |
| 1 | 14 (31.1) | 21 (40.4) |  |
| 2 | 23 (51.1) | 17 (32.7) |  |
| 3 | 7 (15.6) | 9 (17.3) |  |
| 4 | 1 (2.2) | 5 (9.6) |  |
| Mean PRF score ≥ 3 | 7 (15.6) | 17 (32.7) | 0.062 |

**Table S4 - General outcomes – Alternative definition 3**

SD, standard deviation; IQR, interquartile range; GFR, glomerular filtration rate; RRT, renal replacement therapy; KDIGO, Kidney Disease: Improving Global Outcomes; CKD-EPI, Chronic Kidney Disease Epidemiology Collaboration, RS, renal scintigraphy; PRF, persistent renal failure.

Persistent renal failure defined as follow-up eGFR < 50% of baseline eGFR, irrespective of AKI severity. The sum of the accumulation index and accumulation slope of the two kidneys was considered in these analysis. For the other parameters, the values of the best kidney were considered.

^*^ patients on RRT not included ^**^ missing for 1 patient ^***^ missing for 2 patients ^****^ missing for 3 patients

|  | **Recovery**  **(N=20)** | **PRF**  **(N=30)** | **P value** |
| --- | --- | --- | --- |
| **Calculated RS parameters, median (IQR)** |  |  |  |
| Accumulation index^*^ | 7.2 (2.9) | 6.8 (2.9) | 0.3 |
| Elimination index^**^ | 0.8 (0.2) | 0.8 (0.3) | 0.9 |
| Accumulation slope^**^ | 0.4 (0.4) | 0.2 (0.3) | 0.14 |
| **Calculated RS activity ratio, median (IQR)** |  |  |  |
| 3^rd^/2^nd^ minute activity ratio | 1.1 (0.1) | 1.1 (0) | 0.067 |
| 4^th^/2^nd^ minute activity ratio | 1.2 (0.1) | 1.2 (0.1) | 0.5 |
| 6^th^/2^nd^ minute activity ratio | 1.3 (0.3) | 1.3 (0.2) | 0.5 |
| **Calculated RS activity slope, median (IQR)** |  |  |  |
| 3^rd^/2^nd^ minute activity slope | 837.0 (568.3) | 413.5 (503.2) | 0.016 |
| 4^th^/2^nd^ minute activity slope | 619.5 (456.3) | 431.2 (490.3) | 0.3 |
| 6^th^/2^nd^ minute activity slope | 507.6 (482.6) | 347.4 (450.1) | 0.4 |
| **Renal recovery score, n (%)** |  |  | 0.4 |
| Examiner 1 | 2 (10.0) | 5 (16.7) |  |
| 1 | 9 (45.0) | 9 (30.0) |  |
| 2 | 6 (30.0) | 6 (20.0) |  |
| 3 | 3 (15.0) | 10 (33.3) |  |
| 4 |  |  |  |
| Examiner 2 |  |  | 0.5 |
| 1 | 7 (35.0) | 10 (33.3) |  |
| 2 | 9 (45.0) | 11 (36.7) |  |
| 3 | 4 (20.0) | 5 (16.7) |  |
| 4 | 0 (0.0) | 4 (13.3) |  |
| Mean PRF score ≥ 3 | 5 (25.0) | 11 (36.7) | 0.5 |

**Table S5 - General outcomes – Renal replacement therapy (RRT) subgroup**

SD, standard deviation; IQR, interquartile range; GFR, glomerular filtration rate; RRT, renal replacement therapy; KDIGO, Kidney Disease: Improving Global Outcomes; CKD-EPI, Chronic Kidney Disease Epidemiology Collaboration, RS, renal scintigraphy; PRF, persistent renal failure.

Persistent renal failure was defined as ongoing need for RRT at the time of follow-up. The sum of the accumulation index and accumulation slope of the two kidneys was considered in these analysis. For the other parameters, the values of the best kidney were considered.

^*^missing for 1 patients ^**^ missing for 2 patients

|  |  |  | **2.5–97.5% CI** | |  |
| --- | --- | --- | --- | --- | --- |
|  | **n patients** | **odds ratio** | **lower** | **upper** | **P value** |
| **Alternative definition 1** |  |  |  |  |  |
| **Standard model** |  |  |  |  |  |
| + Total accumulation index | 88 | 0.94 | 0.80 | 1.08 | 0.4 |
| + Maximal elimination index | 87 | 1.4 | 0.29 | 6.78 | 0.7 |
| + Total accumulation slope | 86 | 0.36 | 0.07 | 1.47 | 0.19 |
| + Mean PRF score ≥ 3 | 89 | 3.01 | 0.97 | 10.8 | 0.069 |
| + 3^rd^/2^nd^ minute activity ratio | 89 | 6.4x10^-4^ | 1.7x10^-7^ | 0.49 | 0.059 |
| + 3^rd^/2^nd^ minute activity slope | 89 | 0.999 | 0.998 | 1 | 0.039 |
| **Alternative definition 2** |  |  |  |  |  |
| **Standard model** |  |  |  |  |  |
| + Total accumulation index | 88 | 0.90 | 0.77 | 1.05 | 0.2 |
| + Maximal elimination index | 87 | 1.33 | 0.24 | 7.27 | 0.7 |
| + Total accumulation slope | 86 | 0.33 | 0.06 | 1.46 | 0.18 |
| + Mean PRF score ≥ 3 | 89 | 5.5 | 1.41 | 29.2 | 0.024 |
| + 3^rd^/2^nd^ minute activity ratio | 89 | 1.5x10^-4^ | 1.2x10^-8^ | 0.33 | 0.048 |
| + 3^rd^/2^nd^ minute activity slope | 89 | 0.999 | 0.998 | 1 | 0.01 |
| **Alternative definition 3** |  |  |  |  |  |
| **Standard model** |  |  |  |  |  |
| + Total accumulation index | 88 | 0.92 | 0.78 | 1.08 | 0.3 |
| + Maximal elimination index | 87 | 0.42 | 0.06 | 2.87 | 0.4 |
| + Total accumulation slope | 86 | 0.69 | 0.12 | 3.49 | 0.7 |
| + Mean PRF score ≥ 3 | 89 | 1.96 | 0.55 | 8.34 | 0.3 |
| + 3^rd^/2^nd^ minute activity ratio | 89 | 1.8x10^-2^ | 3.1x10^-6^ | 69.4 | 0.3 |
| + 3^rd^/2^nd^ minute activity slope | 89 | 0.999 | 0.998 | 1 | 0.2 |

**Table S6 – Multivariable analyses for Persistent renal failure prediction – Alternative definitions**

PRF, persistent renal failure.

The relation between renal scintigraphy parameters and persistent renal failure were explored in a multivariate logistic regression model for all alternative definitions. Variables included in the standard model were Age, presence of hypertension, presence of type 2 Diabetes, acute kidney injury stage according to the KDIGO guidelines and Baseline renal clearance according to the Chronic Kidney Disease Epidemiology Collaboration (CKD-EPI) equation. The sum of the accumulation index and accumulation slope of the two kidneys was considered in these analysis. For the other parameters, the values of the best kidney were considered. Number of patients included in each analysis is presented on second column (total and percentage out of the 103 patients in the alternative definitions)

Alternative definition 1: Persistent renal failure defined as <25% recovery in estimated GFR within 425 days of RS or as the need for renal replacement therapy.

Alternative definition 2: Persistent renal failure defined as severe CKD (GFR < 30 mL/min/1.73m2) on follow-up, irrespective of baseline function.

Alternative definition 3: Persistent renal failure defined as follow-up eGFR < 50% of baseline eGFR, irrespective of AKI severity.

|  | **Area under curve** | **Best cut-off**  **(for specificity)** | **Sensitivity** | **Specificity** | **PPV** | **NPV** |
| --- | --- | --- | --- | --- | --- | --- |
| **Conventional RS parameters, median (IQR)** |  |  |  |  |  |  |
| Accumulation index^*^ | 0.62 (0.5,0.73) | 4.53 (3,6.15) | 0.19 (0.04,0.52) | 0.89 (0.85,0.95) | 0.67 (0.33,0.87) | 0.48 (0.39,0.64) |
| Elimination index^**^ | 0.48 (0.36,0.59) | 1.33 (1.09,1.54) | 0.12 (0.02,0.37) | 0.88 (0.85,0.96) | 0.55 (0.25,0.82) | 0.45 (0.36,0.57) |
| Accumulation slope^***^ | 0.62 (0.51,0.73) | 0.2 (0.08,0.31) | 0.22 (0.08,0.51) | 0.86 (0.85,0.95) | 0.65 (0.45,0.86) | 0.48 (0.38,0.63) |
| **Calculated RS activity ratio, median (IQR)** |  |  |  |  |  |  |
| 3^rd^/2^nd^ minute activity ratio | 0.7 (0.59,0.79) | 1.1 (1.05,1.13) | 0.38 (0.13,0.62) | 0.86 (0.85,0.94) | 0.77 (0.59,0.89) | 0.54 (0.41,0.67) |
| 4^th^/2^nd^ minute activity ratio | 0.67 (0.56,0.78) | 1.14 (1.06,1.18) | 0.34 (0.14,0.54) | 0.86 (0.85,0.94) | 0.75 (0.6,0.89) | 0.52 (0.41,0.65) |
| 6^th^/2^nd^ minute activity ratio | 0.66 (0.56,0.77) | 1.21 (1.1,1.26) | 0.42 (0.2,0.58) | 0.86 (0.85,0.95) | 0.79 (0.68,0.91) | 0.55 (0.43,0.68) |
| **Calculated RS activity slope, median (IQR)** |  |  |  |  |  |  |
| 3^rd^/2^nd^ minute activity slope | 0.72 (0.61,0.82) | 617 (195,753.5) | 0.49 (0.14,0.72) | 0.86 (0.85,0.95) | 0.81 (0.6,0.91) | 0.58 (0.42,0.75) |
| 4^th^/2^nd^ minute activity slope | 0.71 (0.6,0.81) | 502.5 (146.5,601.5) | 0.51 (0.14,0.66) | 0.86 (0.85,0.93) | 0.82 (0.61,0.91) | 0.59 (0.42,0.71) |
| 6^th^/2^nd^ minute activity slope | 0.69 (0.58,0.79) | 250.5 (105.83,351.75) | 0.42 (0.21,0.61) | 0.86 (0.85,0.94) | 0.79 (0.67,0.9) | 0.55 (0.42,0.69) |
| **Mean PRF score ≥ 3** | 0.62 (0.54,0.71) | 1 (1,1) | 0.36 (0.23,0.49) | 0.89 (0.79,0.97) | 0.79 (0.61,0.95) | 0.53 (0.42,0.65) |

**Table S7 - Diagnostic performance of Clinical score and RS derived parameters – Alternative definition 1**

IQR, interquartile range; PPV, Predictive positive value; NPV, Predictive Negative value; RS, renal scintigraphy, PRF persistent renal failure.

Table of computed sensitivity, specificity, area under curve (with binomial 95% confidence intervals), positive predictive value and negative predictive value (with bootstraps 95% confidence intervals). Cut-off points were performed with fixed specificity above 0.85. The sum of the accumulation index and accumulation slope of the two kidneys was considered in these analysis. For the other parameters, the values of the best kidney were considered.

Persistent renal failure defined as <25% recovery in estimated GFR within 425 days of RS or as the need for renal replacement therapy.

^*^ missing for 1 patient ^**^ missing for 2 patients ^***^missing for 3 patients

|  | **Area under curve** | **Best cut-off**  **(for specificity)** | **Sensitivity** | **Specificity** | **PPV** | **NPV** |
| --- | --- | --- | --- | --- | --- | --- |
| **Conventional RS parameters, median (IQR)** |  |  |  |  |  |  |
| Accumulation index^*^ | 0.65 (0.54,0.77) | 5.5 (4.03,6.15) | 0.39 (0.11,0.52) | 0.85 (0.85,0.95) | 0.79 (0.6,0.91) | 0.49 (0.36,0.61) |
| Elimination index^**^ | 0.52 (0.41,0.64) | 0.64 (0.5,0.71) | 0.21 (0.04,0.35) | 0.9 (0.85,0.95) | 0.75 (0.33,0.9) | 0.44 (0.32,0.55) |
| Accumulation slope^***^ | 0.66 (0.53,0.77) | 0.21 (0.12,0.39) | 0.31 (0.09,0.59) | 0.85 (0.85,0.96) | 0.74 (0.55,0.9) | 0.47 (0.36,0.62) |
| **Calculated RS activity ratio, median (IQR)** |  |  |  |  |  |  |
| 3^rd^/2^nd^ minute activity ratio | 0.69 (0.57,0.79) | 1.1 (1.04,1.13) | 0.34 (0.11,0.59) | 0.85 (0.85,0.94) | 0.77 (0.57,0.91) | 0.47 (0.35,0.63) |
| 4^th^/2^nd^ minute activity ratio | 0.64 (0.53,0.76) | 1.12 (1.05,1.18) | 0.22 (0.08,0.47) | 0.85 (0.85,0.97) | 0.68 (0.5,0.89) | 0.43 (0.33,0.56) |
| 6^th^/2^nd^ minute activity ratio | 0.68 (0.57,0.78) | 1.21 (1,1.28) | 0.4 (0.05,0.59) | 0.85 (0.85,0.93) | 0.79 (0.43,0.9) | 0.49 (0.34,0.63) |
| **Calculated RS activity slope, median (IQR)** |  |  |  |  |  |  |
| 3^rd^/2^nd^ minute activity slope | 0.76 (0.66,0.85) | 715 (195,815) | 0.55 (0.15,0.74) | 0.85 (0.85,0.95) | 0.84 (0.68,0.93) | 0.57 (0.37,0.71) |
| 4^th^/2^nd^ minute activity slope | 0.71 (0.6,0.81) | 414.5 (146.5,601.5) | 0.4 (0.15,0.63) | 0.85 (0.85,0.93) | 0.79 (0.64,0.91) | 0.49 (0.37,0.66) |
| 6^th^/2^nd^ minute activity slope | 0.71 (0.59,0.81) | 250.5 (15,448.5) | 0.4 (0.05,0.64) | 0.85 (0.85,0.95) | 0.79 (0.4,0.91) | 0.49 (0.34,0.65) |
| **Mean PRF score ≥ 3** | 0.63 (0.55,0.71) | 1 (1,1) | 0.36 (0.24,0.49) | 0.9 (0.79,0.98) | 0.84 (0.68,0.96) | 0.49 (0.38,0.61) |

**Table S8 - Diagnostic performance of Clinical score and RS derived parameters – Alternative definition 2**

IQR, interquartile range; PPV, Predictive positive value; NPV, Predictive Negative value; RS, renal scintigraphy, PRF persistent renal failure.

Table of computed sensitivity, specificity, area under curve (with binomial 95% confidence intervals), positive predictive value and negative predictive value (with bootstraps 95% confidence intervals). Cut-off points were performed with fixed specificity above 0.85. The sum of the accumulation index and accumulation slope of the two kidneys was considered in these analysis. For the other parameters, the values of the best kidney were considered.

Persistent renal failure defined as severe CKD (GFR < 30 mL/min/1.73m2) on follow-up, irrespective of baseline function.

^*^ missing for 1 patient ^**^ missing for 2 patients ^***^missing for 3 patients

|  | **Area under curve** | **Best cut-off**  **(for specificity)** | **Sensitivity** | **Specificity** | **PPV** | **NPV** |
| --- | --- | --- | --- | --- | --- | --- |
| **Conventional RS parameters, median (IQR)** |  |  |  |  |  |  |
| Accumulation index^*^ | 0.58 (0.46,0.69) | 4.42 (3,5.55) | 0.16 (0.04,0.42) | 0.87 (0.85,0.95) | 0.57 (0.29,0.83) | 0.48 (0.37,0.6) |
| Elimination index^**^ | 0.61 (0.5,0.71) | 0.68 (0.63,0.84) | 0.29 (0.16,0.49) | 0.89 (0.85,0.96) | 0.75 (0.58,0.91) | 0.52 (0.41,0.63) |
| Accumulation slope^***^ | 0.59 (0.47,0.7) | 0.2 (0.08,0.31) | 0.22 (0.08,0.52) | 0.86 (0.85,0.95) | 0.65 (0.45,0.86) | 0.49 (0.41,0.65) |
| **Calculated RS activity ratio, median (IQR)** |  |  |  |  |  |  |
| 3^rd^/2^nd^ minute activity ratio | 0.67 (0.57,0.77) | 1.1 (1.07,1.13) | 0.38 (0.21,0.6) | 0.91 (0.85,0.96) | 0.83 (0.67,0.93) | 0.56 (0.44,0.69) |
| 4^th^/2^nd^ minute activity ratio | 0.62 (0.5,0.73) | 1.13 (1.06,1.17) | 0.29 (0.14,0.48) | 0.87 (0.85,0.95) | 0.71 (0.57,0.88) | 0.51 (0.41,0.64) |
| 6^th^/2^nd^ minute activity ratio | 0.6 (0.49,0.71) | 1.13 (1.08,1.24) | 0.23 (0.13,0.51) | 0.89 (0.85,0.94) | 0.71 (0.55,0.87) | 0.5 (0.4,0.65) |
| **Calculated RS activity slope, median (IQR)** |  |  |  |  |  |  |
| 3^rd^/2^nd^ minute activity slope | 0.71 (0.6,0.81) | 617 (240.86,772) | 0.5 (0.22,0.75) | 0.87 (0.85,0.93) | 0.81 (0.67,0.91) | 0.6 (0.44,0.77) |
| 4^th^/2^nd^ minute activity slope | 0.66 (0.54,0.76) | 414.5 (185.5,572.5) | 0.42 (0.22,0.59) | 0.87 (0.85,0.93) | 0.79 (0.67,0.9) | 0.57 (0.44,0.68) |
| 6^th^/2^nd^ minute activity slope | 0.61 (0.5,0.72) | 162.75 (85.25,344.25) | 0.27 (0.12,0.49) | 0.87 (0.85,0.95) | 0.7 (0.54,0.88) | 0.51 (0.41,0.65) |
| **Mean PRF score ≥ 3** | 0.59 (0.5,0.67) | 1 (1,1) | 0.33 (0.21,0.47) | 0.84 (0.73,0.94) | 0.71 (0.5,0.88) | 0.52 (0.41,0.63) |

**Table S9 - Diagnostic performance of Clinical score and RS derived parameters – Alternative definition 3**

IQR, interquartile range; PPV, Predictive positive value; NPV, Predictive Negative value; RS, renal scintigraphy, PRF persistent renal failure.

Table of computed sensitivity, specificity, area under curve (with binomial 95% confidence intervals), positive predictive value and negative predictive value (with bootstraps 95% confidence intervals). Cut-off points were performed with fixed specificity above 0.85. The sum of the accumulation index and accumulation slope of the two kidneys was considered in these analysis. For the other parameters, the values of the best kidney were considered.

Persistent renal failure defined as follow-up eGFR < 50% of baseline eGFR, irrespective of AKI severity.

^*^ missing for 1 patient ^**^ missing for 2 patients ^***^missing for 3 patients

|  | **Area under curve** | **Best cut-off**  **(for specificity)** | **Sensitivity** | **Specificity** | **PPV** | **NPV** |
| --- | --- | --- | --- | --- | --- | --- |
| **Conventional RS parameters, median (IQR)** |  |  |  |  |  |  |
| Accumulation index^*^ | 0.59 (0.42,0.74) | 5.5 (3.2,6.27) | 0.38 (0.09,0.55) | 0.85 (0.85,1) | 0.79 (0.6,1) | 0.49 (0.32,0.63) |
| Elimination index^**^ | 0.51 (0.34,0.67) | 1.21 (0.83,1.3) | 0.1 (0.03,0.54) | 0.95 (0.47,1) | 0.75 (0.33,1) | 0.41 (0.27,0.57) |
| Accumulation slope^**^ | 0.63 (0.45,0.79) | 0.13 (0.02,0.31) | 0.21 (0.04,0.65) | 0.9 (0.85,1) | 0.75 (0.4,1) | 0.45 (0.32,0.66) |
| **Calculated RS activity ratio, median (IQR)** |  |  |  |  |  |  |
| 3^rd^/2^nd^ minute activity ratio | 0.65 (0.48,0.82) | 1.04 (0.97,1.12) | 0.23 (0.04,0.71) | 0.85 (0.72,1) | 0.7 (0.43,1) | 0.42 (0.29,0.67) |
| 4^th^/2^nd^ minute activity ratio | 0.55 (0.39,0.72) | 1.06 (0.98,1.2) | 0.17 (0.03,0.6) | 0.9 (0.47,1) | 0.71 (0.33,1) | 0.42 (0.27,0.59) |
| 6^th^/2^nd^ minute activity ratio | 0.55 (0.38,0.73) | 1.05 (1.03,1.32) | 0.13 (0.06,0.64) | 0.85 (0.38,0.95) | 0.57 (0.4,0.92) | 0.4 (0.25,0.61) |
| **Calculated RS activity slope, median (IQR)** |  |  |  |  |  |  |
| 3^rd^/2^nd^ minute activity slope | 0.7 (0.53,0.85) | 195 (-144,715) | 0.27 (0.04,0.84) | 0.9 (0.85,1) | 0.8 (0.5,1) | 0.45 (0.3,0.77) |
| 4^th^/2^nd^ minute activity slope | 0.58 (0.4,0.75) | 127.5 (-42.5,591.11) | 0.2 (0.03,0.62) | 0.85 (0.5,1) | 0.67 (0.33,1) | 0.41 (0.29,0.65) |
| 6^th^/2^nd^ minute activity slope | 0.57 (0.4,0.75) | 24 (15,488) | 0.07 (0.03,0.64) | 0.9 (0.44,1) | 0.5 (0.25,1) | 0.39 (0.27,0.62) |
| **Mean PRF score ≥ 3** | 0.56 (0.43,0.68) | 1 (1,1) | 0.37 (0.2,0.55) | 0.75 (0.53,0.94) | 0.69 (0.43,0.92) | 0.44 (0.29,0.61) |

**Table S10 - Diagnostic performance of Clinical score and RS derived parameters – Renal replacement therapy (RRT) subgroup**

IQR, interquartile range; PPV, Predictive positive value; NPV, Predictive Negative value; RS, renal scintigraphy, PRF persistent renal failure.

Table of computed sensitivity, specificity, area under curve (with binomial 95% confidence intervals), positive predictive value and negative predictive value (with bootstraps 95% confidence intervals). Cut-off points were performed with fixed specificity above 0.85. The sum of the accumulation index and accumulation slope of the two kidneys was considered in these analysis. For the other parameters, the values of the best kidney were considered.

For patients who were receiving RRT at the time of RS, persistent renal failure was defined as ongoing need for RRT at the time of follow-up.

^*^ missing for 1 patient ^**^ missing for 2 patients


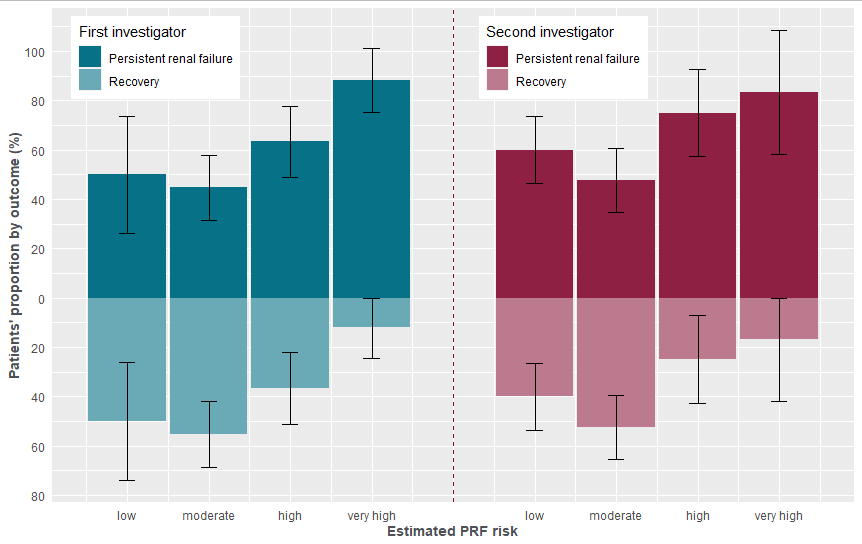


**Figure S1 – Patients’ proportions by estimated PRF risk and by outcome**

PRF, persistent renal failure. Two nuclear medicine specialists (Investigator 1 and 2) were asked to estimate PRF risk using a pre-established scale (1: low estimated PRF risk, 2: moderate estimated PRF risk, 3: high estimated PRF risk, 4: very high estimated PRF risk). The graphic presents the proportion of patients classified by estimated PRF risk and outcome, for each investigator.


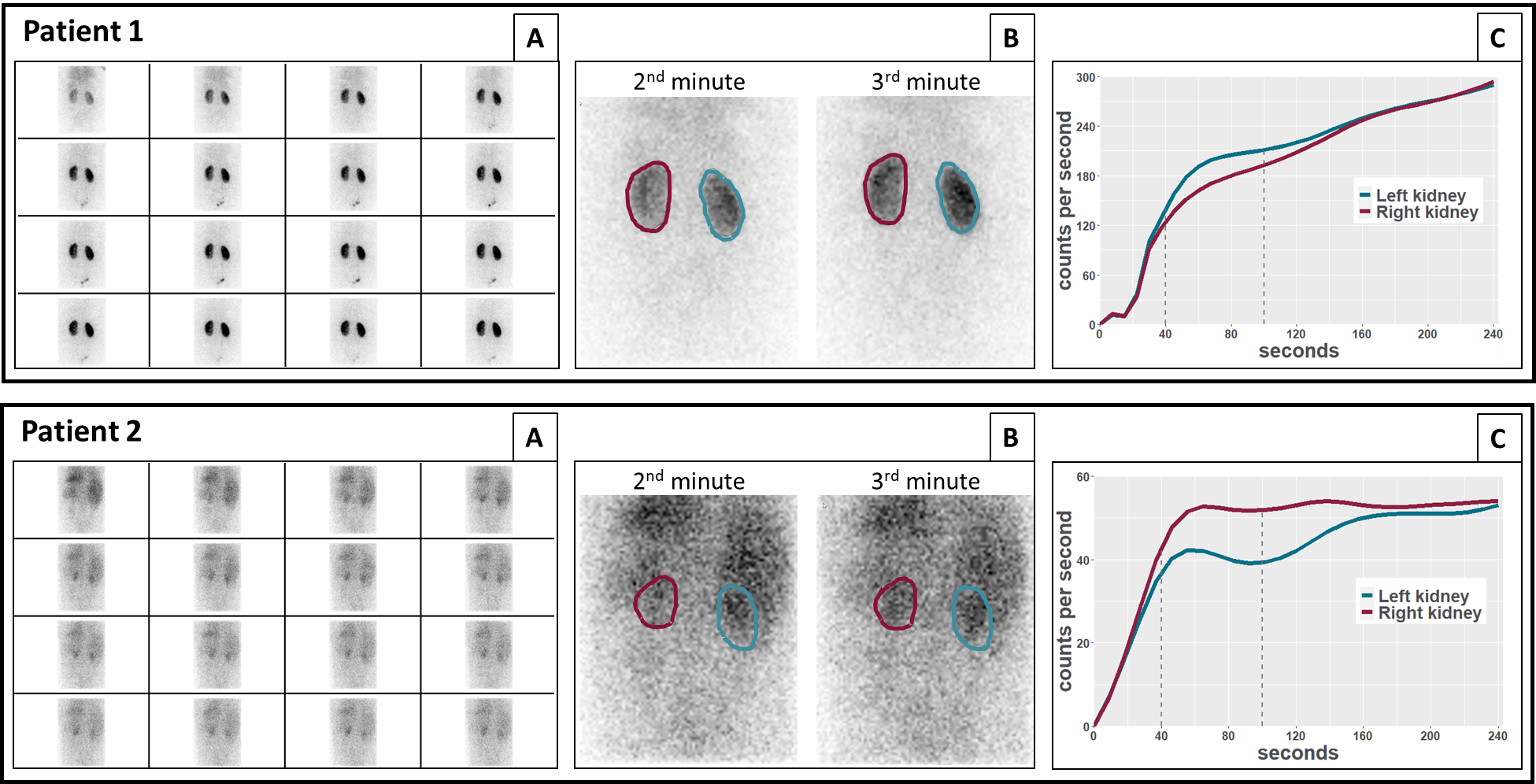


**Figure S2 – Renal scintigraphy images, regions of interest and time activity curves in two patients**

Patient 1 was a 56 years old patient presenting with AKI who recovered renal function at one year (recovery group). Patient 2 was an 18 years old patient who did not recover renal function after AKI (Persistent renal failure group). Panel A shows one-minute images starting 30 seconds after iode-123-hippuran injection, reflecting parenchymal radiotracer accumulation over time. Panel B shows one-minute images at 2 and 3 minutes with associated regions of interest (ROI) from which the activity ratios and slopes are calculated. Panel C shows time activity curves for each kidney. The accumulation index is calculated based on the area under the curve between 40 seconds and 100 seconds post-injection.
